# Supplementary material for: An extracellular matrix protein promotes anillin-dependent processes in the Caenorhabditis elegans germline
Source: Life Sci Alliance. 2019 Apr 15;2(2):e201800152. doi: 10.26508/lsa.201800152 (PMC6467243; doi:10.26508/lsa.201800152)
Supplement: Supplementary file 15 [file LSA-2018-00152_TableS1.doc]

**Table S1 *C.elegans*** strains used in this study

| **Strains** | **Marker** | **Genotype** | **Cross** |
| --- | --- | --- | --- |
| **N2** | - | Ancestral N2 Bristol strain; “wild-type” | - |
| **SA245** | mCherry::HIS | *unc-119(ed3); tjls57 [pie-1::mCherry::his-48; unc-119(+)]* | - |
| **OD58** | GFP::PH | *unc-119(ed3) III; ltIs38 [pAA1; pie-1::GFP::PH(PLC1delta1); unc-119 (+)]* | - |
|
| **LP162** | NMY-2::GFP | *cp13[nmy-2::gfp + LoxP]* | - |
|
| **OD70** | mCherry::PH | *unc-119(ed3) III; ltIs44[pAA173; pie-1::mCherry::PH(PLC1delta1); unc-119 (+)] V.* | - |
|
| **BT24** | GFP::HIM-4 | *rhIs23 [GFP::him-4] III* | - |
| **CB1266** | *him-4(e1266)* | *him-4(e1266) X* | - |
| **UM208** | GFP::ANI-2;mCherry::PH | *unc-119(ed3)III;Itls81[Ppie-1::gfp-TEV-Stag::ani-2;unc-119(+)];ltls44[Ppie-1::mCherry::PH(PLC1delta1);unc-119(+)]IV* |  |
| **MG535** | GFP::PH; mCherry::HIS | - | OD58 × SA245 |
| **YC0158** | GFP::ANI-1 | *yc01[gfp::ani-1 + LoxP]* | - |
| **YC0130** | GFP::HIM-4; mCherry::HIS | - | BT24 × SA245 |
| **YC0159** | GFP::HIM-4; mCherry::HIS; mCherry::PH | - | OD70 × YC0130 |
| **YC0032** | NMY-2::GFP; mCherry::HIS | - | LP162 × SA245 |
| **YC0186** | GFP::ZEN-4 | *Yc02[gfp::zen-4 + LoxP]* |  |
| **YC0207** | GFP::ZEN-4; mCherry::HIS |  | YC0186 × SA245 |
